# Laparoscopic subtotal cholecystectomy for difficult gallbladder management: A single-center study

**DOI:** 10.6026/973206300221065

**Published:** 2026-02-28

**Authors:** Vishal Patil, Pramod Verma, Dheeraj Kain, Rishi Kumar Athya, Nitin Patta, Akansha Sahare, Sachin Parmar

**Affiliations:** 1 Department of Gastrointestinal Surgery, Bhopal Memorial Hospital and Research centre, Bhopal, Madhya Pradesh, India; 2 Department of Surgical Oncology, Bhopal Memorial Hospital and Research centre, Bhopal, Madhya Pradesh, India; 3 Department of Obstetrics and Gynaecology, Mahaveer Medical College, Bhopal, Madhya Pradesh, India; 4 Department of Community Medicine, V.K.S. Government Medical College, Neemuch, Madhya Pradesh, India

**Keywords:** Laparoscopic subtotal cholecystectomy, difficult gallbladder, Bailout procedure

## Abstract

In cases of severe inflammation, fibrosis, or distorted anatomy, achieving a "Critical View of Safety" during laparoscopic cholecystectomy is often impossible, which significantly increases the risk of catastrophic bile duct injury. The laparoscopic subtotal cholecystectomy is a very important bailout operation in that complete cholecystectomy cannot be performed in a challenging injury of the gallbladder due to extreme inflammation or the absence of anatomy. This retrospective BMHRC Bhopal (January 2024-January 2025) retrospective study looked at 55 patients with a combination of indications, such as chronic calculouscholecystitis, Mirizzi syndrome, gallbladder perforation and post-ERCP situation, with a mean of 78.5 ± 24.3 minutes of operative time, 4.2 ± 2.8 days of hospital stay and a complication rate of 12 per cent, 2 per cent Modified laparoscopic subtotal cholecystectomy is a safe and effective compromised surgical procedure where avoidance of great bile duct injury is a priority and where operative variables, postoperative complications and minimized final outcome are satisfactory with 8.7 ± 3.2 months at the mean follow-up.

## Background:

Cholecystectomy is a common surgery on the abdomen that is done all over the world [1]. The operation may be technically challenging due to dense adhesions in Calot's triangle. It has been suggested that these situations can be handled by switching to open surgery or subtotal cholecystectomy [2, 3]. The word "partial" is no longer used and subtotal cholecystectomies are now split into two groups: "fenestrating" and "reconstituting." Subtotal reconstituting cholecystectomy closes off the bottom of the gallbladder, which lowers the risk of postoperative fistula. However, it leaves behind a gallbladder, which could cause symptomatic cholecystolithiasis to come back [4]. The Tokyo Guidelines stress the importance of using bailout procedures-subtotal cholecystectomy, fundus-first dissection, or conversion- when it is not safe to identify cystic structures. This shows that patient safety is more important than finishing a total cholecystectomy in difficult surgical fields. Guideline-informed safe steps include recognizing the "difficult gallbladder," thinking about bailout early on and using structured intraoperative algorithms to avoid biliary injury [5]. This puts Laparoscopic subtotal cholecystectomy (LSC) within standardized safe cholecystectomy frameworks for both acute and chronic inflammation. In this case, the "modified" subtotal cholecystectomy-usually meaning center-specific improvements like planned mucosectomy, closing the remnant with stitches or loops when possible, selective internal drainage and regular stone clearance from Hartmann's pouch-tries to reduce bile leak while stopping remnant stone disease by using the general fenestrating/reconstituting principles and adapting them to the local expertise and resources [6].

Ultimately, LSC is an important skill for surgeons who are dealing with complicated biliary inflammation. It is a controlled compromise that avoids major biliary injury and fits with safety-first principles that are supported by modern guidance [7]. A systematic review of 2,166 laparoscopic surgeries found that common reasons for surgery were acute cholecystitis. Early outcomes showed very low rates of bile duct injury and death, but high rates of bile leaks that usually go away with endoscopic or percutaneous management. Late events included incisional hernia, symptomatic or remnant stones and a small number of patients needing a completion cholecystectomy [6]. The choice of subtotal cholecystectomy technique should be based on the surgeon's comfort and experience with different techniques and intraoperative findings, even though the reconstituted procedure may be preferred when possible. Long-term follow-up studies are still needed to fully understand how each method fits into a general surgeon's toolkit for treating complex gallbladder (GB) patients [8]. Laparoscopic modified subtotal cholecystectomy is a safe bailout procedure for difficult gallbladder cases where complete cholecystectomy risks biliary injury. Therefore, it is of interest to evaluate its technical feasibility, perioperative outcomes and complications, emphasizing patient safety over procedural completion in complex biliary inflammation.

## Methods and Materials:

## Study design and setting:

This retrospective observational study was executed at the Department of Gastro-Surgery, Bhopal Memorial Hospital and Research Centre (BMHRC), Bhopal, India, over duration of one year, from January 2024 to January 2025. The study received registration from BMHRC Bhopal and obtained ethical approval from the Institutional Ethics Committee (IEC) of BMHRC Bhopal. The study population comprised adult patients who underwent laparoscopic cholecystectomy at BMHRC Bhopal during the designated study period. The study encompassed both elective and emergency cholecystectomies. We got patient data from the hospital records of people who had laparoscopic cholecystectomy, focusing on those who needed modified subtotal cholecystectomy for difficult gallbladder conditions.

## Criteria for Inclusion and Exclusion:

## Criteria for inclusion:

[1] Adult patients (both male and female) aged 18 to 80 years.

[2] Patients who had laparoscopic cholecystectomy, whether it was planned or done in an emergency.

## Exclusion criteria:

[1] Patients who are either less than 18 years old or more than 80 years old.

[2] Patients who are critically ill or have unstable blood flow and are not fit for surgery.

[3] Patients with known coagulopathy or hemorrhagic disorders.

## Data collection:

We looked back at hospital records to get information on patients who had laparoscopic cholecystectomy during the study period.

## We wrote down and looked at the following parameters:

[1] Demographic Information: Age, sex, body mass index (BMI) and comorbidities (*e.g.*, diabetes mellitus, hypertension).

[2] Preoperative tests: hemoglobin levels, tests of kidney and liver function, random blood sugar (RBS), chest X-ray, electrocardiogram (ECG), abdominal ultrasound (USG), contrast-enhanced computed tomography (CECT) of the abdomen and magnetic resonance cholangiopancreatography (MRCP) if done.

[3] Operative Details: Reasons for modified subtotal cholecystectomy (*e.g.*, severe inflammation, fibrosis, unclear anatomy at Calot's triangle, dense adhesions), length of the surgery, problems that happened during the surgery (*e.g.*, bleeding, bile spillage, gallbladder perforation) and where to put the drain.

## Postoperative data:

Length of hospital stay, immediate postoperative complications (within 24 hours), early complications (1-7 days) and late complications (beyond 7 days). We also kept track of follow-up data, such as the resolution of symptoms, the return to normal activities, long-term complications and patient satisfaction. 5. Follow-up: All patients were followed up on a regular basis to look for early and late complications and to see how well they were doing. Follow-up data were gathered at one, three and six months, with the last follow-up taking place at an average of 8.7 ± 3.2 months.

## Statistical analysis:

We used descriptive statistics to look at the data we collected. For continuous variables, we used mean ± standard deviation (SD) and for categorical variables, we used frequency (percentage). We looked at the median length of stay in the hospital, the median operative time and the rates of complications (both during and after surgery). They also looked at the overall rates of illness and death. We used the right software (like SPSS, version 25) to do statistical analysis and show the results.

## Ethical considerations:

The Institutional Ethics Committee (IEC) of BMHRC Bhopal gave this study the go-ahead. Patient consent was not necessary due to the retrospective nature of the study. But all of the patient data was made anonymous and handled in a way that followed ethical rules for using medical data.

## Limitations of the study:

Being a retrospective observational study, it has some problems, like relying on hospital records that may not show all the variables or details. Moreover, being a single-center study, the results may lack full generalizability to other contexts.

## Results and Discussion:

During the study period, 55 patients underwent laparoscopic subtotal cholecystectomy (LSTC), with two conversions to open surgery and a cohort characterized by a mean age of 47.8 ± 15.2 years and a female predominance (61.3% female), with indications including chronic calculouscholecystitis, emphysema of the gallbladder, gallbladder perforation, Mirizzi syndrome, post-ERCP for CBD calculi and gangrenous gallbladder (Table 1). Preoperatively, patients had a mean BMI of 26.4 ± 4.1 kg/m^2^, with comorbidity burdens of diabetes (25.3%) and hypertension (28.0%), ASA grades distributed as 52.0% I, 43.3% II and 4.7% III and presenting complaints dominated by right upper quadrant pain (100%) with frequent nausea/vomiting (78.7%) (Table 2). Intraoperatively, among 53 analyzed cases, the mean operative time was 78.5 ± 24.3 minutes; LSTC was primarily undertaken for severe inflammation/fibrosis (59.3%) and unclear Calot’s anatomy (50.7%), with additional findings of acute inflammation, gangrene, dense adhesions, emphysema, contracted gallbladder and Mirizzi syndrome; stump closure was achieved using endoloops or intracorporeal absorbable sutures and four patients required postoperative ERCP (two for bile leak and two for CBD stricture) (Table 3). Postoperatively, the mean hospital stay was 4.2 ± 2.8 days, immediate complications within 24 hours included bleeding (5.3%) and bile leak (8.0%), overall postoperative events comprised bile leak (12%), CBD stricture (2%) and port site hernia (3%), with 4-5 readmissions for pain or collection; mean follow-up was 8.7 ± 3.2 months and patient satisfaction averaged 8.4 ± 1.6 on a 10-point scale (Table 4).

The present study's conversion rate, operative time, hospital stay and morbidity profile are broadly concordant with contemporary evidence supporting laparoscopic subtotal cholecystectomy (LSTC) as an effective bailout strategy when the critical view of safety cannot be achieved, with trade-offs that include a higher incidence of bile leak and selective need for postoperative ERCP compared with total cholecystectomy, as documented by Elshaer *et al.* [7], Koo *et al.* [9], Caroline *et al.* [10] and Ramírez-Giraldo *et al.* [11] This morbidity profile is further substantiated by Deng *et al.* [12], who reported a 25.8% bile leak rate in fenestrating LSTC, yet achieved zero bile duct injuries, reinforcing the procedure's safety profile despite higher minor complications.The cohort's mean operative time of 78.5 minutes and short length of stay are within reported ranges from single-center series and meta-analyses described by Elshaer *et al.* [7], Caroline *et al.* [10] and Shin *et al.* [13] in 2016. These findings align closely with Haldeniya *et al.* [15], who reported a mean operative time of 86.2 minutes and a hospital stay of 2.5 days in a tertiary care setting. However, the influence of severe inflammation on operative duration is variable; Alshammary *et al.* [14] recently reported a significantly longer mean operative time of 164 minutes and a length of stay of 9.14 days in a high-complexity cohort, highlighting the impact of fibrotic adhesions on surgical workflow. The indications in this series-severe inflammation or fibrosis, unclear Calot's anatomy, Mirizzi syndrome, perforation and post-ERCP scenarios-mirror those most frequently cited in systematic reviews by Elshaer *et al.* [7] and RamÍrez-Giraldo *et al.* [11] as triggers for subtotal techniques. Haldeniya *et al.* [15] further validate these indications, noting that 50% of their LSTC cases were performed for difficult pathology following ERCP. The observed bile leak rate with targeted postoperative ERCP parallels pooled data from Elshaer *et al.* [7], Koo *et al.* [9], Caroline *et al.* [10] and Deng *et al.* [12] showing that while subtotal cholecystectomy reduces the risk of common bile duct injury relative to forcing completion in hostile anatomy, it carries a predictable increase in bile leakage, subhepatic collections and reinterventions that are typically manageable with minimally invasive approaches. Management of Mirizzi syndrome within the cohort aligns with reports by Kimura *et al.* [16] and Saavedra *et al.* [17] advocating LSTC to mitigate ductal injury risk in high-hazard anatomy, particularly when secure stump closure techniques are employed, with outcomes consistent with published series on laparoscopic subtotal strategies in Mirizzi and other complex presentations. Overall, the convergence of findings across operative metrics, complication patterns and postoperative pathways supports LSTC as a safe, feasible option that prioritizes avoidance of bile duct injury while accepting a known, manageable bile leak profile in carefully selected difficult cholecystectomy cases, as demonstrated by Elshaer *et al.* [7], Koo *et al.* [9], Shin *et al.* [13] and Alshammary *et al.* [14].

## Conclusion:

Laparoscopic subtotal cholecystectomy is a secure salvage procedure for challenging gallbladder cases that effectively reduces the risk of major bile duct injury when the critical view of safety is impractical. By prioritizing patient safety over procedural completion, this controlled surgical compromise leads to satisfactory long-term results and high patient satisfaction. Mastering this modified technique is essential for general surgeons dealing with complex biliary inflammation to ensure safe and efficacious outcomes.

## Figures and Tables

**Table 1:** Overview of cases (Laparoscopic Subtotal Cholecystectomy)

**Variable**	**Values (n=55)**
Total Cases	55
Converted to Open Surgery	2
Indications for LSTC	Chronic Calculus Cholecystitis, Emphysema of Gall Bladder, Gall Bladder Perforation, Mirizzi Syndrome, Post-ERCP for CBD Calculi, Gangrenous Gall Bladder
Average Age (mean ± SD)	47.8 ± 15.2 years
Gender (Male/Female)	38.7% Male, 61.3% Female

**Table 2:** Preoperative characteristics

**Variable**	**Values (n=55)**
BMI (mean ± SD)	26.4 ± 4.1 kg/m^2^
Comorbidities	25.3% Diabetes, 28.0% Hypertension
ASA Grade (I/II/III)	52.0% ASA I, 43.3% ASA II, 4.7% ASA III
Presenting Symptoms	100% Right upper quadrant pain, 78.7% Nausea/Vomiting, etc.

**Table 3:** Intraoperative details

**Variable**	**Values (n=53)**
Operative Time (mean ± SD)	78.5 ± 24.3 minutes
Indications for LSTC	59.3% Severe inflammation/fibrosis, 50.7% Unclear anatomy at Calot's triangle, etc.
Intraoperative Findings	Acute Inflammation, Gangrenous Gall Bladder, Dense Adhesions, Emphysema, Contracted Gall Bladder, Mirizzi Syndrome
Stump Closure Method	Endoloop or Intracorporeal Absorbable Suture
Need for ERCP Post-Op	4 patients (2 for bile leak, 2 for CBD stricture)

**Table 4:** Postoperative outcomes and complications

**Variable**	**Values (n=53)**
Hospital Stay (mean ± SD)	4.2 ± 2.8 days
Immediate Complications (≤24 hrs)	5.3% Bleeding, 8.0% Bile leak, etc.
Postoperative Complications	12% Bile leak, 2% CBD stricture, 3% Port site hernia, etc.
Readmissions for Pain/Collection	4-5 patients
Follow-up Duration (mean ± SD)	8.7 ± 3.2 months
Patient Satisfaction (1-10)	Mean score: 8.4 ± 1.6
